# The impact of COVID lockdown on glycaemic control in paediatric patients with type 1 diabetes: A systematic review and meta-analysis of 22 observational studies

**DOI:** 10.3389/fendo.2022.1069559

**Published:** 2022-11-30

**Authors:** Yanping Han, Yuqing Chen, Chenyu Sun, Zhen Zhou

**Affiliations:** ^1^ Department of Endocrinology and Metabolism, Anhui Provincial Children’s Hospital, Hefei, Anhui, China; ^2^ AMITA Health Saint Joseph Hospital Chicago, University of Illinois College of Medicine, Chicago, IL, United States; ^3^ Menzies Institute for Medical Research, University of Tasmania, Hobart, TAS, Australia

**Keywords:** type 1 diabetes, glucose, COVID, lockdown, paediatrics

## Abstract

**Introduction:**

The COVID lockdown has posted a great challenge to paediatric patients with type 1 diabetes (T1D) and their caregivers on the disease management. This systematic review and meta-analysis sought to compare the glycaemic control among paediatric patients with T1D (aged under 18 years) pre- during, and post-lockdown period.

**Methods and materials:**

We did a systematic search of three databases (PubMed, Embase, and the WHO COVID‐19 Global literature) for the literature published between 1 Jan 2019 to 10 Sep 2022. Studies meeting the following inclusion criteria were eligible for this study: (1) a COVID-19 related study; (2) inclusion of children aged 18 years old or under with established T1D; (3) comparing the outcomes of interest during or after the COVID lockdown with that before the lockdown. Study endpoints included mean difference (MD) in HbA1c, blood glucose, time in range (TIR, 70-180 mg/dl), time above range (TAR, >180mg/dl), time below range (TBR,<70mg/dl) and glucose variability (coefficient of variation [CV]) between pre-lockdown and during lockdown and/or between pre- and post-lockdown period. The MD and its corresponding 95% CI of each endpoint were pooled using random-effect model considering the potential between-study heterogeneity in COVID restrictions and T1D management.

**Results:**

Initial search identified 4488 records and 22 studies with 2106 paediatric patients with T1D were included in the final analysis. Compared with pre-lockdown period, blood glucose was significantly decreased by 0.11 mmol/L (95%CI: -0.18, -0.04) during lockdown period and by 0.42 mmol/L (95%CI: -0.73, -0.11) after lockdown. The improvement was also found for TIR, TAR, TBR, and CV during and post-lockdown (all p values<0.05) except for the post-lockdown TBR (*p* =0.35). No significant change in HbA1c was observed during and post- lockdown period when compared with the pre-lockdown value. There was moderate to high between-study heterogeneity for most of the analyses.

**Conclusion:**

Compared with pre-lockdown period, there was significant improvement in T1D paediatric patients’ glucose metrics during and post-lockdown. The underlying reasons for this positive impact warrant further investigation to inform future paediatric diabetes management.

**Systematic Review Registration:**

https://www.crd.york.ac.uk/PROSPERO/, identifier CRD42022359213.

## Introduction

COVID-19 lockdown was implemented by many countries during the outbreak to minimise the SARS-CoV-2 transmission. The lockdown restrictions including temporary closure of non-essential activities and businesses including schools and enforcement of limited outdoor activities and social interactions have significantly changed people’s lifestyle and daily routine patterns. Type 1 diabetes (T1D) is an immune disease that is generally found and diagnosed in childhood. Paediatric patients with T1D were likely to be affected by the COVID lockdown remarkably due to the COVID-associated changing in habits and dietary patterns, reduced physical activity, and mental problems due to restrictions. In addition, the lockdown can increase patients’ difficulty in seeking timely medical healthcare and the likelihood of a shortage of insulin (1, 2).

Previous works studying the impact of COVID lockdown on disease management in TID paediatric patients has yielded mixed findings, with some reporting a better glucose control during and post lockdown period (3, 4) and others reporting the opposite (5, 6). The robustness of evidence from historical studies was universally limited by a small sample size, thus a more comprehensive analysis is warranted to generate robust evidence to inform the impact of lockdown on glucose control among T1D paediatric patients during the pandemic. Based on this we performed a systematic review and meta-analysis of observational studies by comparing the glucose control in T1D patients aged under 18 years between pre-lockdown and lockdown, and between pre-lockdown and post-lockdown period.

## Methods and materials

This systematic review and meta-analysis followed the Preferred Reporting Items for Systematic reviews and Meta-Analysis statement (PRISMA). Ethical approval was exempted for this analysis as we only used aggregate data extracted from previous publications. The study protocol was registered at the PROSPERO (CRD42022359213).

### Search strategy

We did a systematic search of three databases which included PubMed, Embase, and the World Health Organization (WHO) COVID‐19 Global literature on coronavirus disease from 2019 when COVID-19 firstly emerged to 10 Sep 2022. The following keywords were used for searching in various combinations: Type 1, diabetes, COVID, coronavirus and SARS-Cov-2. Studies that fulfilled all the following inclusion criteria were eligible for this study: (1) a COVID-19 related study; (2) inclusion of children aged 18 years old or under with established T1D; (3) comparing the outcomes of interest during or after the COVID lockdown with that before the lockdown. Studies that included new-onset T1D, animal studies and case reports were excluded. We made no restriction on language. To minimise the reporting bias, we also manually searched the relevant articles and screened the papers in reference lists of the included studies. Titles and abstracts screening was undertaken by two reviewers (YPH, ZZ) in an independent manner. Discrepancies were revolved by discussion between the two reviewers, if the inconsistency still present, suggestions from a third reviewer (YQC) were sought.

### Data extraction and quality assessment

The data were extracted and collated by ZZ using a standardized Excel spreadsheet and audited by YPH. The quality of each included study was evaluated using the Newcastle-Ottawa Scale for cohort studies (7). The summed score ≤3 was rated as having poor quality.

### Endpoint

The endpoints included mean difference in HbA1c, blood glucose, time in range (TIR, percent of time 70-180 mg/dl), time above range (TAR, precent of time >180mg/dl), time below range (TBR, percent of time<70mg/dl) and glucose variability (coefficient of variation [CV]) between pre-lockdown and during lockdown and between pre- and post-lockdown period.

### Data analysis

If a study only reported median and interquartile (IQR), the data were then transferred to mean and standard deviation (SD) using Hozo’s method (8). As the majority of studies only reported mean values and SD for each glycaemic metric before (T0), during (T1), and after-lockdown (T2) period and did not report the mean difference (MD) and its corresponding SD between T0 and T1 or T2, we estimated the SD for the MD by using the formula: $SD=\sqrt{SD_{T0}^{2}+SD_{T1/T2}^{2}-2*r*SD_{T0}*SD_{T1/T2}}$ (9); where r denotes the correlation between pre-lockdown and follow-up glycaemic data and was given an approximate value of 0.5 which is considered a conservative estimate when using the change scores from baseline (10). The 95% confidence interval (CI) was calculated using SD. We then pooled the MD and the corresponding 95% CI for each individual outcome by using pre-specified random-effect models with restricted maximum likelihood estimation considering the potential heterogeneity in COVID restrictions and T1D management across countries. A subgroup analysis was performed by limiting to studies including more than 80% of T1D patients using continuous glucose monitoring/flash glucose monitoring (CGM/FGM). Between-study heterogeneity was measured by *I* ^2^ statistics, with I^2^ value of 50% or higher suggesting high heterogeneity. Potential publication bias was checked with Egger’s test and the visualisation of funnel plots when there are 10 or more studies. Leave-one-out sensitivity analysis was conducted for each individual outcome to detect small study effect by excluding one study per time iteratively.

A p value<0.05 was considered statistically significant. All meta-analyses were performed in Stata 17 (Stata Corp, College Station, TX, USA).

## Results

### Literature search

The initial search on three databases identified 4488 studies. After removing 692 duplicates, 3796 articles were screened against title and abstract, with 3726 articles being further excluded. Full text of 48 articles were read, and among these, 22 studies with 2106 paediatric patients with T1D were included in the final analysis. Eleven studies compared glucose control between the lockdown and pre-lockdown period (3, 4, 11–19); eight studies compared post-lockdown with pre-lockdown period (5, 6, 20–26); three studies compared both lockdown and post-lockdown with pre-lockdown period (26–28); In most studies the glycaemic data were collected *via* CGM or FGM. The characteristics of the included studies are presented in **Table 1**. The flow chart of study selection is shown in **Supplementary Figure 1**.

**Table 1:** Baseline characteristics of included studies.

study	COVID-context	Sample size	Country	Continent	Age (mean)	Diabetes duration	Male (%)	CSII (%)	MCI (%)	CGM/FGM
Alsalman et al. (2022) (11)	Before and during lockdown	164	Saudi Arabia	Asia	12.5 ± 3.7	NA	45.1	12.2	73.8	NA
Brener et al.(2020) (12)	Before and during lockdown	102	Israel	Asia	11.2 ± 3.8	4.2 ± 3.8	52.9	NA	NA	NA
Cheng et al. (2021) (20)	Before and after lockdown	93	Malaysia	Asia	11.1 ± 3.5	4.6 ± 3.1	44.0	6.5	93.5	1
Christoforidis et al. (2020) (13)	Before and during lockdown	34	Greece	Europe	11.4 ± 4.5	5.1 ± 3.4	47.1	100	0	100%
Cognigni et al. (2021) (21)	Before and after lockdown	50	Italy	Europe	14.8 ± 1.3	7.1 ± 1.4	50.0	62.0	38	84%
Conejero et al. (2022) (14)	Before and during lockdown	80	Spain	Europe	12.6 ± 3.3	5.9 ± 3.9	56.0	66.2	33.8	90.2%
Dalmazi et al. (2020) (4)	Before and during lockdown	54	Italy	Europe	10.7 ()?	5.5 ()?	59.0	0%	42.6	100%
Di Riso et al. (2021) (22)	Before and after lockdown	71	Italy	Europe	11.0 ± 2.3	5.7 ± 3.0	53.4	47.9	52.1	91.5%
Duarte et al. (2022) (6)	Before and after lockdown	100	Portugal	Europe	12.5 ± 4.0	7 ± 4	59.0	100	0	NA
Elhenawy et al.(2021) (5)	Before and after lockdown	115	Egypt	Africa	0-18	>6months	53.0	5.2	94.8	13.9%
Garza et al. (2022) (27)	Before, during and after lockdown	157	Spain	Europe	12.6 ± 3.2	6.7 ± 3.7	57.1	41	59.0	88.5%
Hakonen et al. (2022) (15)	Before and during lockdown	245	Finland	Europe	11.1 ± 2.9	5.4 ± 3.3	53.0	63.0	33.0	100%
Lombardo et al. (2021) (28)	Before, during and after lockdown	85	Italy	Europe	11.5 ± 3.7	5.2 ± 3.3	51.6	77.6	22.4	100%
Marigliano et al.(2021) (23)	Before and after lockdown	233	Italy	Europe	13.9 ± 4.4	6.9 ± 4.4	55.7	38.6	61.4	85%
Minuto et al.(2021) (16)	Before and during lockdown	107	Italy	Europe	6-<18	NA	NA	NA	NA	NA
Nwosu et al. (2021) (24)	Before and after lockdown	110	USA	North America	14.8 ± 4.9	6.3 ± 4.3	51.8	41.8	58.0	59.1%
Predieri et al.(2020) (17)	Before and during lockdown	62	Italy	Europe	11.1 ± 4.4	4.9 ± 4.2	50.0	46.8	53.2	100%
Schiaffini et al. (2020) (18)	Before and during lockdown	22	Italy	Europe	8.7 ± 1.9	>1 year	63.6	100	0	100%
Tinti et al. (2021) (19)	Before and during lockdown	66	Italy	Europe	11.6 ± 4.5	4.5 ± 3	46.0	55	45	100%
Tornese et al. (2020) (3)	Before and during lockdown	13	Italy	Europe	13.8 ± 1.2	5.9 ± 1.9	61.5	100	0	100%
Turan et al. (2022) (25)	Before and after lockdown	100	Turkey	Asia	14.7 ± 3.4	7.2 ± 3.6	45.0	NA	NA	NA
Wu et al. (2021) (26)	Before, during and after lockdown	43	China	Asia	7.5 ± 3.2	1.2 ± 0.3	NA	69.8	30.2	100%

CSII, continuous subcutaneous infusion insulin; MCI, multiple continuous injection; CGM/FGM, continuous glucose monitoring/flash glucose monitoring; NA, not available.

### Risk of bias

The risk of bias of included studies are summarised in **Supplementary Table 1**. All studies were deemed at high quality (summed score ≥5). We gave one star to all studies in term of the comparability due to the paired data were used (comparing the repeated measures of a same individual and thus the confounder adjustment is not needed).

### Impact of lockdown on HbA1c

**Figure 1:**
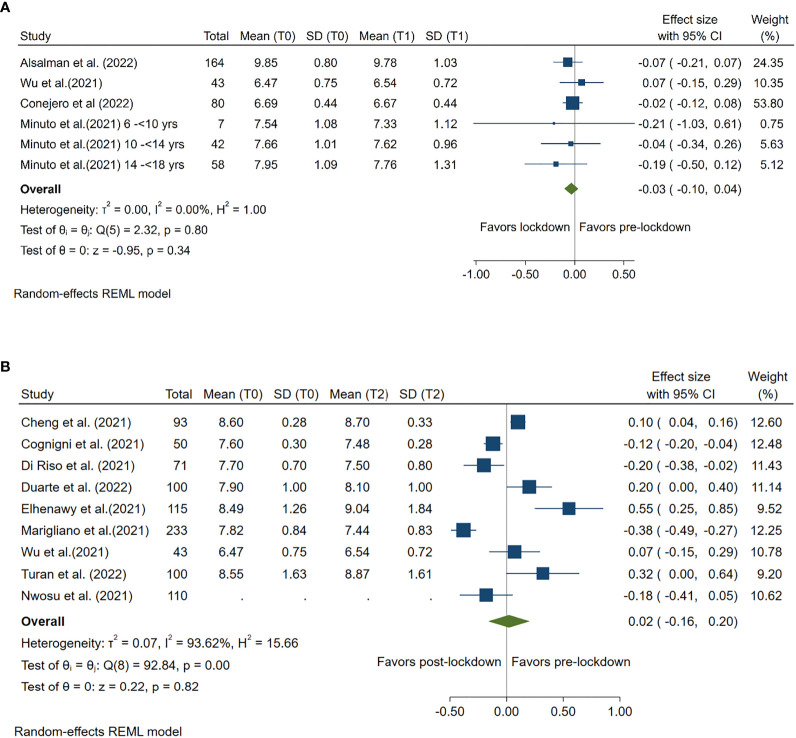
Forest plot of HbA1c. **(A)** Lockdown (T1) versus pre-lockdown (T0). **(B)** Post-lockdown (T2) versus pre-lockdown (T0).

Four studies compared mean HbA1c levels pre- and during lockdown and 9 studies compared mean HbA1c levels pre- and post-lockdown. Compared with pre-lockdown period, no significant reduction or increase in HbA1c was found for either lockdown (Summary MD: -0.03%; 95%CI: -0.10%, 0.04%, *p* =0.34) or post-lockdown period (Summary MD: 0.02%; 95%CI: -0.16%, 0.20%, *p* =0.82). (**Figure 1**)

### Impact of lockdown on blood glucose

**Figure 2:**
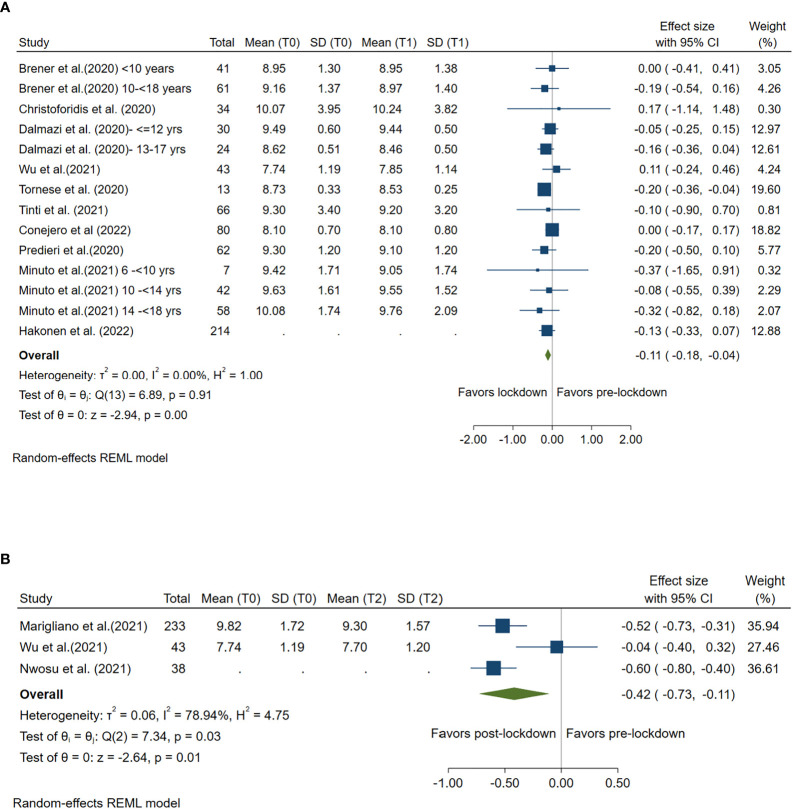
Forest plot of mean blood glucose. **(A)** Lockdown (T1) versus pre-lockdown (T0). **(B)** Post-lockdown (T2) versus pre-lockdown (T0).

Ten studies compared mean blood glucose levels pre- and during lockdown, and 3 studies compared mean HbA1c levels pre- and post-lockdown. Compared with pre-lockdown period, blood glucose was decreased by 0.11 mmol/L (95%CI: -0.18, -0.04) during lockdown period and by 0.42 mmol/L (95%CI: -0.73, -0.11) after lockdown. Both reductions were statistically significant (*p<*0.001 and *p* =0.01, respectively). (**Figure 2**)

### Impact of lockdown on time in range

**Figure 3:**
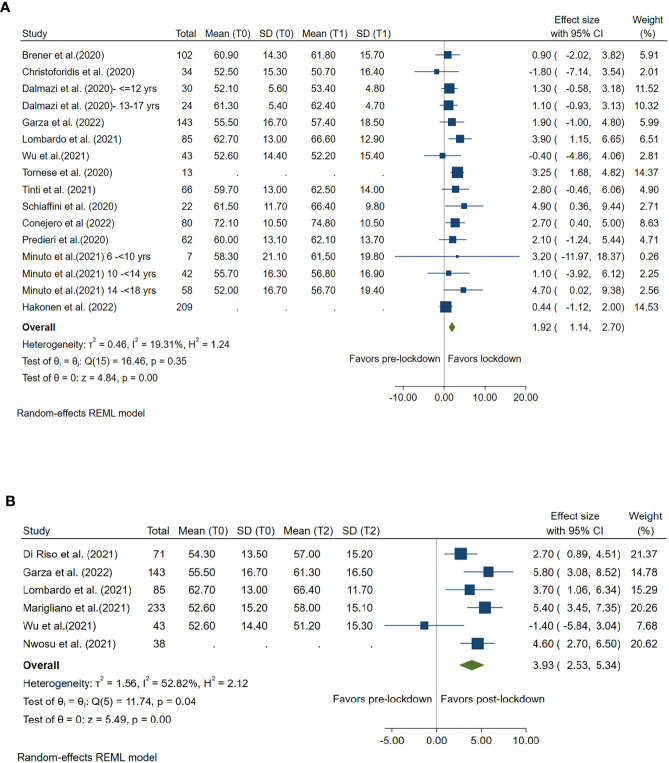
Forest plot of time in range (glucose 70-80mg/dl). **(A)** Lockdown (T1) versus pre-lockdown (T0). **(B)** Post-lockdown (T2) versus pre-lockdown (T0).

TIR 70-180 mg/dl during lockdown versus pre-lockdown was reported by 13 studies and during post-lockdown versus pre-lockdown was reported by 6 studies. Compared with pre-lockdown period, TIR 70-180 mg/dl was significantly increased by 1.92% (95%CI: 1.14%, 2.70%, *p<*0.001) during lockdown period and by 3.93% (95%CI: 2.53%, 5.34%, *p<*0.001) after lockdown. (**Figure 3**)

### Impact of lockdown on time above range

**Figure 4:**
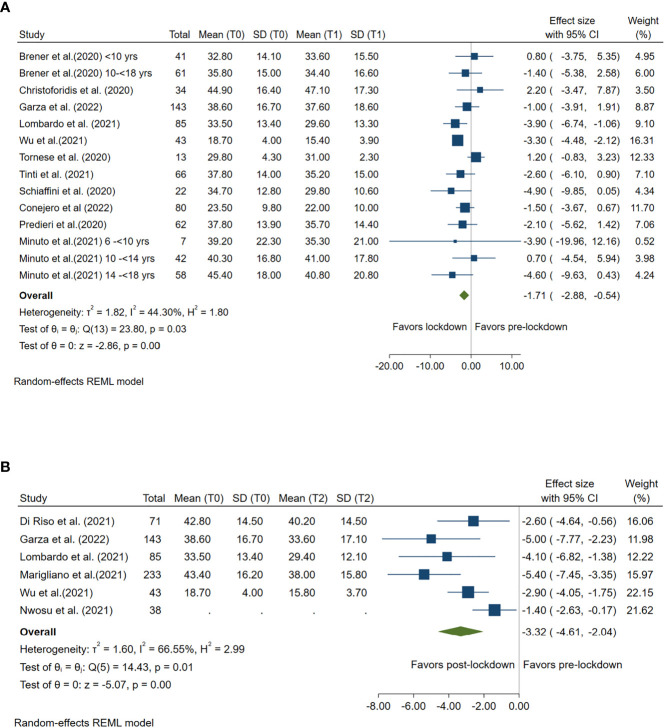
Forest plot of time above range (glucose≥180mg/dl). **(A)** Lockdown (T1) versus pre-lockdown (T0). **(B)** Post-lockdown (T2) versus pre-lockdown (T0).

TAR ≥180 mg/dl was compared between lockdown and pre-lockdown period by 11 studies and was compared between post- and pre-lockdown period by 6 studies. TAR was significantly reduced in both lockdown (Summary MD: -1.71%, 95%CI: -2.88%, -0.54%, *p<*0.001) and post-lockdown period (Summary MD: -3.32%, 95%CI: -4.61%, -2.04%, *p<*0.001) when compared with the TAR during pre-lockdown period. (**Figure 4**)

### Impact of lockdown on time below range

**Figure 5:**
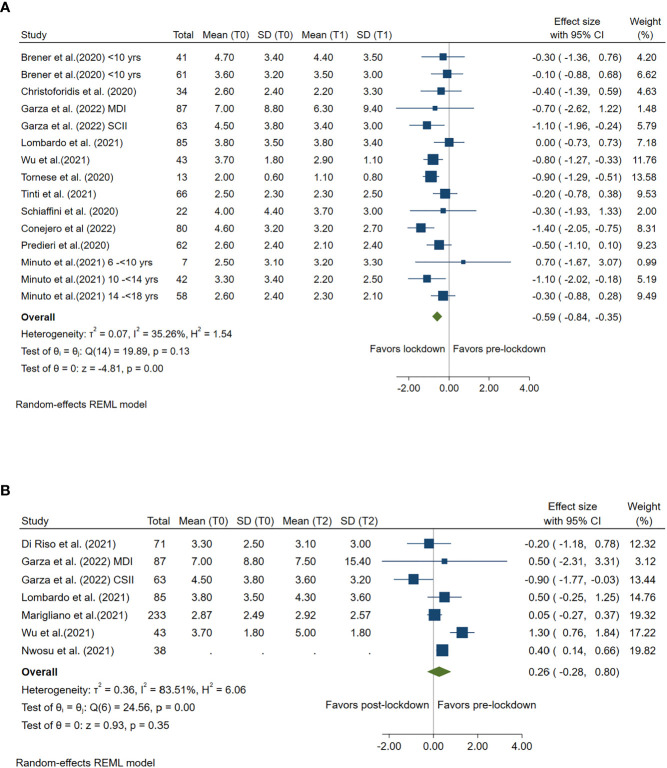
Forest plot of time below range (glucose≤70mg/dl). **(A)** Lockdown (T1) versus pre-lockdown (T0). **(B)** Post-lockdown (T2) versus pre-lockdown (T0).

TBR ≤70 mg/dl was compared between lockdown and pre-lockdown period by 11 studies and was compared between post- and pre-lockdown period by 6 studies. Compared with pre-lockdown period, TBR was significantly reduced during lockdown (Summary MD: -0.59%, 95%CI: -0.84%, -0.35%, *p<*0.001), but no change was found during post-lockdown period (Summary MD: 0.26%, 95%CI: -0.28%, 0.80%, *p* =0.35). (**Figure 5**)

### Impact of lockdown on coefficient of variation [%CV]

**Figure 6:**
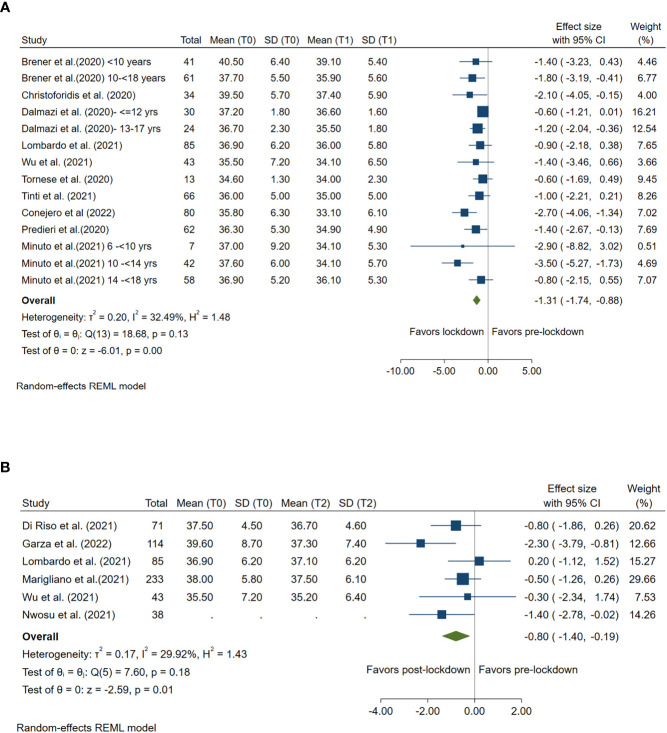
Forest plot of CV. **(A)** Lockdown (T1) versus pre-lockdown (T0). **(B)** Post-lockdown (T2) versus pre-lockdown (T0).

Ten studies compared CV pre- and during lockdown and 6 studies compared CV pre- and post-lockdown. A significant reduction in CV was found during both lockdown (Summary MD: -1.31%; 95%CI: -1.74%, -0.88%, *p<*0.001) and post-lockdown period (Summary MD: -0.80%; 95%CI: -1.40%, -0.19%, *p* =0.01) when compared with the CV level in pre-lockdown period. (**Figure 6**)

### Between-study heterogeneity

High between-study heterogeneity (I^2^≥50%) was seen for all analyses compared post-lockdown and pre-lockdown period, except for the analysis for CV. (**Figures 1**–**6**)

### Subgroup analysis

Results from the subgroup analysis including only studies with ≥80% of T1D patients using CGM/FGM were congruent with the main findings except for the loss of statistical significance of the analysis comparing blood glucose pre- and post-lockdown. The between-study heterogeneity was only slightly improved (**Supplementary Figure 2**).

### Publication bias and leave-one-out sensitivity analyses

Egger’s test results are shown in **Supplementary Table 2** and funnel plots of outcomes which were reported by 10 or more studies are shown in **Supplementary Figure 3**. We found a potential publication bias of analysis for the CV between during and pre-lockdown in both egger’s test and funnel plot. However, such potential bias was not confirmed in the leave-one-out analysis (**Supplementary Figure 4**), in which the significant reduction of CV during lockdown was observed in every analysis excluding one single study. Similarly, results for other outcomes in the leave-one-out analyses (**Supplementary Figure 4**) were also consistent with the main results, except for the glucose change from pre-lockdown to post-lockdown, due likely to the insufficient studies (n=2) to have enough power to main the statistical significance observed in the main analysis.

## Discussion

This systematic review and meta-analysis included 22 observational studies with a total of 2106 paediatric patients with T1D. Compared with the period before COVID lockdown, patients had a significantly improved glucose level and other CGM/FGM metrics including TIR, TAR, TBR, and CV during and after the lockdown period, except for the post-lockdown TBR. No significant difference in mean HbA1c was observed between during and pre-lockdown, nor between post- and pre-lockdown. This study provides an important insight into the potential impact of lockdown and confinement in the context of COVID-19 on paediatric patients’ adaptation to T1D and self-disease management.

The positive impact of COVID-19 lockdown on glucose control in paediatric patients with T1D children might be explained by different reasons. First, the lockdown has enabled the parents and caregivers of the paediatric patients to be better involved in their diabetes management. During the lockdown most families were required to work from home or to comply with home confinement, allowing them to spend more time to partake in the glucose monitoring and diabetes care with their kids. A robust body of studies have showed that parental support and supervision is crucial for maintaining paediatric patients’ adherence to regular glucose monitoring and optimal glucose control (29, 30). Also, the COVID pandemic has remarkably accelerated the development of telemedicine that can provide additional benefits onto the standard diabetes care. In the context of COVID-19, more and more paediatric diabetologists and other health providers started to adopt and utilize virtual healthcare (telemedicine visit and consultation *via* video or phone) along with the advanced monitoring equipment (e.g. sensor CGM/FGM use) in their T1D paediatric patients to facilitate diabetes management (31). More studies are warranted to further explore the underlying reasons leading to this positive impact of lockdown, given that the knowledge may be useful for aiding clinicians in making better strategies for achieving an ideal glycaemic control and quality of care in diabetes paediatric patients.

Although the majority of studies found a positive impact of lockdown on T1D management in paediatric patients, few found the worsened glucose control linked to lockdown. There was moderate to high heterogeneity for most of the analyses. The between-study heterogeneity might come from different accessibility to the telemedicine and CGM/FGM devices during the lockdown and from the varying COVID restriction policies across countries and regions, although no noticeable improvement in between-study heterogeneity was seen when limiting the meta-analysis to only studies including sensor CGM/FGM users. Cheng et al (20), who found a moderate increase in HbA1c levels in paediatric T1D patients after the lockdown in Malaysia compared with the pre-lockdown values pointed to that the inconsistency between their results and others might be due to the lack of access to the valid telemedicine, high cost of CGM device and limited medical resource in the place where the study participants came from.

### Study limitations

This meta-analysis was subject to several limitations. First, all included studies are observational studies with a small sample size, therefore the power may be insufficient for some analyses such as HbA1c. Second, moderate to high heterogeneity between studies was observed from the majority of analyses, which may be explained by the different accessibility to the telemedicine/virtual health care across countries. Given most of studies including T1D patients using CGM/FGM that are more costly than the self-monitored glucose measurement, the study results may not be generalized to countries with limited medical resources. Third, the SD of the mean change of values of all outcomes between lockdown/post-lockdown and pre-lockdown were estimated using a formula rather than directly obtained from the literature in which such data were lacking. This may lead to the 95% CI of the pooled MD being less accurate, more likely being wider due to the use of a conservative coefficient (r=0.5) for the correlation between pre-lockdown and follow-up glucose data. Fourth, it is still unclear whether the positive impact of lockdown on glucose control in T1D patients is driven mainly by the lockdown itself or by the greater use of telehealth. More studies are needed to identify the underlying reasons leading to improved glucose control during and post COVID lockdown period thus to inform better clinical strategies for paediatric diabetes management.

## Conclusion

This systematic review and meta-analysis found significantly improved glucose levels and other continuous glucose monitoring metrics among paediatric patients with type 1 diabetes throughout COVID lockdown when compared with the pre-lockdown period. These results are reassuring and suggests that the T1D paediatric patients are coping well with their disease. Whether the positive impact of lockdown on patient’s glucose control is driven mainly by the advancement of telehealth warrants further investigations.

## Data availability statement

The original contributions presented in the study are included in the article/**Supplementary Material**. Further inquiries can be directed to the corresponding author.

## Author contributions

Study design: All authors. Data extraction: YH, ZZ. Data analysis: YH, ZZ. Results interpretation: All authors. Manuscript drafting: YH, ZZ. Critical revision: All authors. All authors contributed to the article and approved the submitted version.

## Conflict of interest

The authors declare that the research was conducted in the absence of any commercial or financial relationships that could be construed as a potential conflict of interest.

## Publisher’s note

All claims expressed in this article are solely those of the authors and do not necessarily represent those of their affiliated organizations, or those of the publisher, the editors and the reviewers. Any product that may be evaluated in this article, or claim that may be made by its manufacturer, is not guaranteed or endorsed by the publisher.
