# Supplementary material for: The impact of COVID lockdown on glycaemic control in paediatric patients with type 1 diabetes: A systematic review and meta-analysis of 22 observational studies
Source: Front Endocrinol (Lausanne). 2022 Nov 30;13:1069559. doi: 10.3389/fendo.2022.1069559 (PMC9748149; doi:10.3389/fendo.2022.1069559)
Supplement: Supplementary file 1 [file DataSheet_1.pdf]

Supplementary Material

Supplementary Table 1. Newcastle-Ottawa Scale for cohort studies.

Study	Selection				Comparability of cohort	Outcome			Total score
	Representativeness of exposed cohort	Selection of non-exposed cohort	Ascertainment of exposure	Outcome not present at baseline		Assessment of outcome	Sufficient follow-up duration	Adequate follow-up	
Alsalman et al. (2022)	★		★		★	★	★	★	6
Brener et al.(2020)	★		★		★	★	★	★	6
Cheng et al. (2021)	★		★		★	★	★	★	6
Christoforidis et al. (2020)	★		★		★	★	★	★	6
Cognigni et al. (2021)	★		★		★	★	★	★	6
Conejero et al (2022)	★		★		★	★	★	★	6
Dalmazi et al. (2020)	★		★		★	★	★	★	6
Di et al. (2021)	★		★		★	★	★	★	6
Duarte et al. (2022)	★		★		★	★	★	★	6
Elhenawy et al.(2021)	★		★		★	Self-reported	★	★	5
Garza et al. (2022)	★		★		★	★	★	★	6
Hakonen et al. (2022)	★		★		★	★	★	★	6
Lombardo et al. (2021)	★		★		★	★	★	★	6

Marigliano et al.(2021)	★		★		★	★	★	★	6
Minuto et al.(2021)	★		★		★	★	★	★	6
Nwosu et al. (2021)	★		★		★	★	★	★	6
Predieri et al.(2020)	★		★		★	★	★	★	6
Schiaffini et al. (2020)	★		★		★	★	★	★	6
Tinti et al. (2021)	★		★		★	★	★	★	6
Tornese et al. (2020)	★		★		★	★	★	★	6
Turan et al. (2022)	★		★		★	★	★	★	6
Wu et al. (2021)	★		★		★	★	★	★	6

Supplementary Table 2. Egger's test.

	P value
Glucose (lockdown vs. pre-lockdown)	0.99
TIR (lockdown vs. pre-lockdown)	0.81
TAR (lockdown vs. pre-lockdown)	0.84
TBR (lockdown vs. pre-lockdown)	0.33
CV (lockdown vs. pre-lockdown)	0.008

Supplementary Fig 1. Flow chart of study selection.

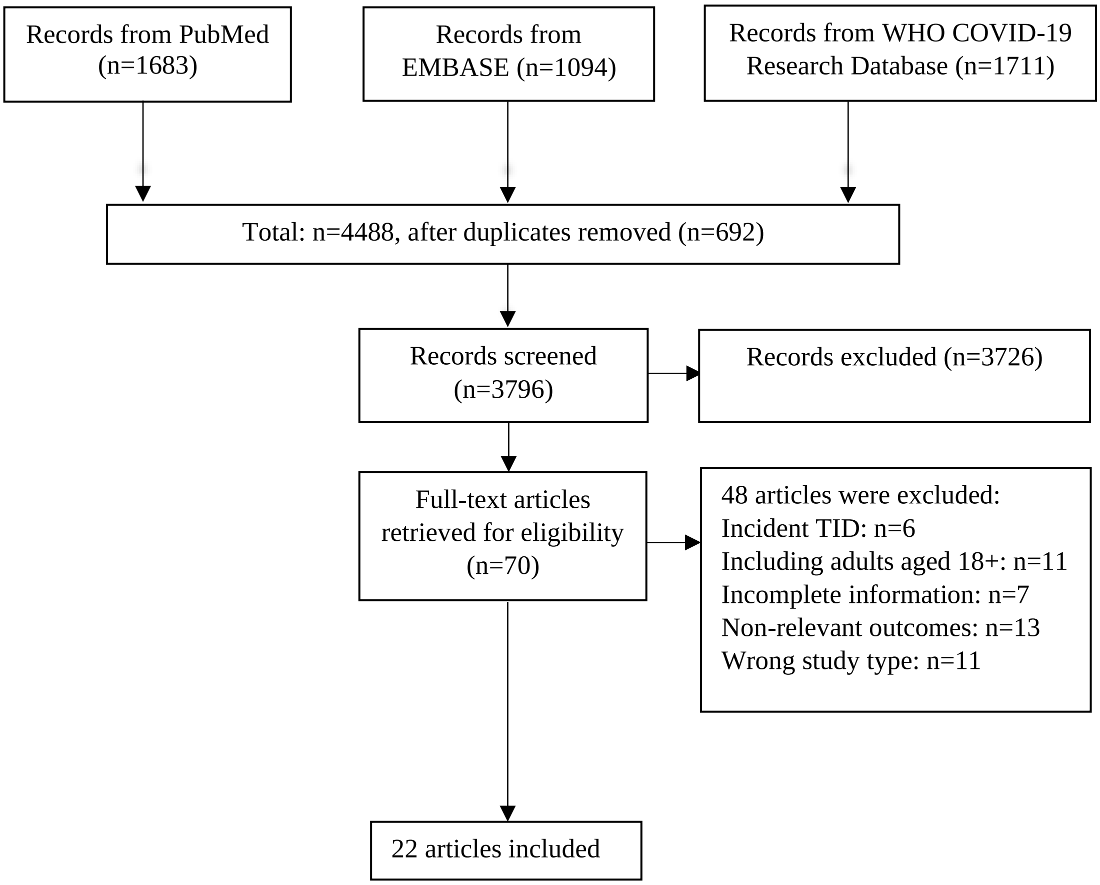

Supplementary Fig 2. Subgroup analysis including only studies with more than 80% of T1D patients using continuous glucose monitoring.

(A) HbA1c (lockdown vs. pre-lockdown)

Study	Total	Mean (T0)	SD (T0)	Mean (T1)	SD (T1)	Effect size with 95% CI	Weight (%)
Wu et al.(2021)	43	6.47	0.75	6.54	0.72	0.07 (-0.15, 0.29)	16.13
Conejero et al (2022)	80	6.69	0.44	6.67	0.44	-0.02 (-0.12, 0.08)	83.87
**Overall**						-0.01 (-0.09, 0.08)	

Heterogeneity: $\tau^2 = 0.00$, $I^2 = 0.00\%$, $H^2 = 1.00$

Test of $\theta_i = \theta_j$: Q(1) = 0.54, p = 0.46

Test of $\theta = 0$: z = -0.12, p = 0.90

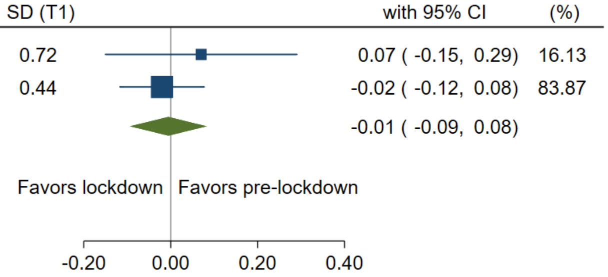

Random-effects REML model

(B) HbA1c (post-lockdown vs. pre-lockdown)

Study	Total	Mean (T0)	SD (T0)	Mean (T2)	SD (T2)	Effect size with 95% CI	Weight (%)
Cognigni et al. (2021)	50	7.60	0.30	7.48	0.28	-0.12 (-0.20, -0.04)	21.42
Di Riso et al. (2021)	71	7.70	0.70	7.50	0.80	-0.20 (-0.38, -0.02)	20.20
Elhenawy et al.(2021)	115	8.49	1.26	9.04	1.84	0.55 (0.25, 0.85)	17.80
Marigliano et al.(2021)	233	7.82	0.84	7.44	0.83	-0.38 (-0.49, -0.27)	21.16
Wu et al.(2021)	43	6.47	0.75	6.54	0.72	0.07 (-0.15, 0.29)	19.41
**Overall**						-0.04 (-0.33, 0.26)	

Heterogeneity: $\tau^2 = 0.10$, $I^2 = 95.17\%$, $H^2 = 20.69$

Test of $\theta_i = \theta_j$: Q(4) = 43.33, p = 0.00

Test of $\theta = 0$: z = -0.23, p = 0.82

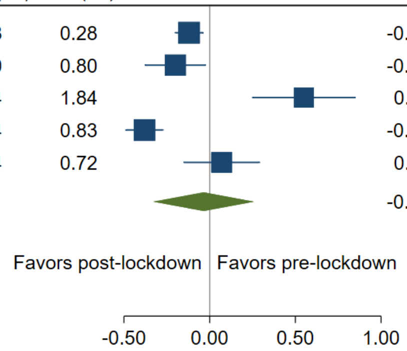

Random-effects REML model

(C) glucose (lockdown vs. pre-lockdown)

Study	Total	Mean (T0)	SD (T0)	Mean (T1)	SD (T1)	Effect size with 95% CI	Weight (%)
Christoforidis et al. (2020)	34	10.07	3.95	10.24	3.82	0.17 (-1.14, 1.48)	0.34
Dalmazi et al. (2020)- <=12 yrs	30	9.49	0.60	9.44	0.50	-0.05 (-0.25, 0.15)	14.74
Dalmazi et al. (2020)- 13-17 yrs	24	8.62	0.51	8.46	0.50	-0.16 (-0.36, 0.04)	14.33
Wu et al.(2021)	43	7.74	1.19	7.85	1.14	0.11 (-0.24, 0.46)	4.82
Tornese et al. (2020)	13	8.73	0.33	8.53	0.25	-0.20 (-0.36, -0.04)	22.28
Tinti et al. (2021)	66	9.30	3.40	9.20	3.20	-0.10 (-0.90, 0.70)	0.92
Conejero et al (2022)	80	8.10	0.70	8.10	0.80	0.00 (-0.17, 0.17)	21.38
Predieri et al.(2020)	62	9.30	1.20	9.10	1.20	-0.20 (-0.50, 0.10)	6.56
Hakonen et al. (2022)	214	.	.	.	.	-0.13 (-0.33, 0.07)	14.63
**Overall**						-0.10 (-0.18, -0.03)	

Heterogeneity: $\tau^2 = 0.00$, $I^2 = 0.00\%$, $H^2 = 1.00$

Test of $\theta_i = \theta_j$: Q(8) = 5.52, p = 0.70

Test of $\theta = 0$: z = -2.61, p = 0.01

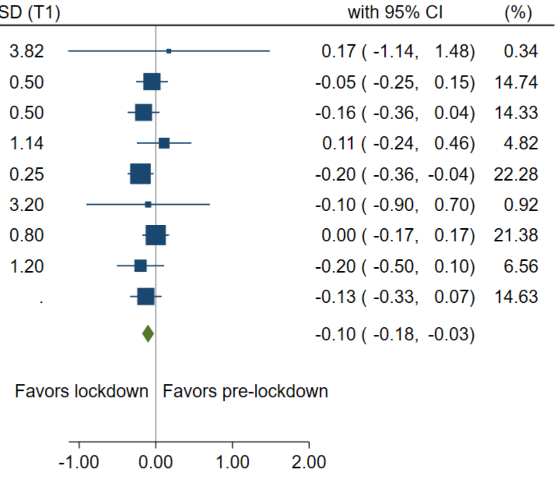

Random-effects REML model

(D) glucose (post-lockdown vs. pre-lockdown)

Study	Total	Mean (T0)	SD (T0)	Mean (T2)	SD (T2)	Effect size with 95% CI	Weight (%)
Marigliano et al.(2021)	233	9.82	1.72	9.30	1.57	-0.52 (-0.73, -0.31)	54.67
Wu et al.(2021)	43	7.74	1.19	7.70	1.20	-0.04 (-0.40, 0.32)	45.33
**Overall**						-0.30 (-0.77, 0.17)	

Heterogeneity: $\tau^2 = 0.09$, $I^2 = 80.51\%$, $H^2 = 5.13$

Test of $\theta_i = \theta_j$: Q(1) = 5.13, p = 0.02

Test of $\theta = 0$: z = -1.27, p = 0.21

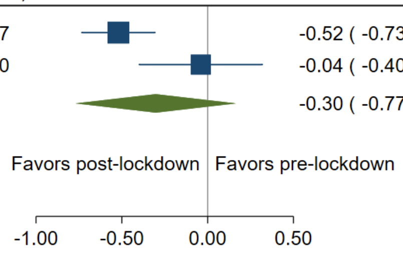

Random-effects REML model

(E) TIR (lockdown vs. pre-lockdown)

Study	Total	Mean (T0)	SD (T0)	Mean (T1)	SD (T1)	Effect size with 95% CI	Weight (%)
Christoforidis et al. (2020)	34	52.50	15.30	50.70	16.40	-1.80 (-7.14, 3.54)	2.36
Dalmazi et al. (2020)- <=12 yrs	30	52.10	5.60	53.40	4.80	1.30 (-0.58, 3.18)	12.82
Dalmazi et al. (2020)- 13-17 yrs	24	61.30	5.40	62.40	4.70	1.10 (-0.93, 3.13)	11.55
Garza et al. (2022)	143	55.50	16.70	57.40	18.50	1.90 (-1.00, 4.80)	6.87
Lombardo et al. (2021)	85	62.70	13.00	66.60	12.90	3.90 (1.15, 6.65)	7.45
Wu et al.(2021)	43	52.60	14.40	52.20	15.40	-0.40 (-4.86, 4.06)	3.28
Tornese et al. (2020)	13	.	.	.	.	3.25 (1.68, 4.82)	15.75
Tinti et al. (2021)	66	59.70	13.00	62.50	14.00	2.80 (-0.46, 6.06)	5.65
Schiaffini et al. (2020)	22	61.50	11.70	66.40	9.80	4.90 (0.36, 9.44)	3.17
Conejero et al (2022)	80	72.10	10.50	74.80	10.50	2.70 (0.40, 5.00)	9.75
Predieri et al.(2020)	62	60.00	13.10	62.10	13.70	2.10 (-1.24, 5.44)	5.44
Hakonen et al. (2022)	209	.	.	.	.	0.44 (-1.12, 2.00)	15.91
**Overall**						1.93 (1.08, 2.78)	

Heterogeneity: $\tau^2 = 0.55$, $I^2 = 25.84\%$, $H^2 = 1.35$

Test of $\theta_i = \theta_j$: Q(11) = 14.51, p = 0.21

Test of $\theta = 0$: z = 4.46, p = 0.00

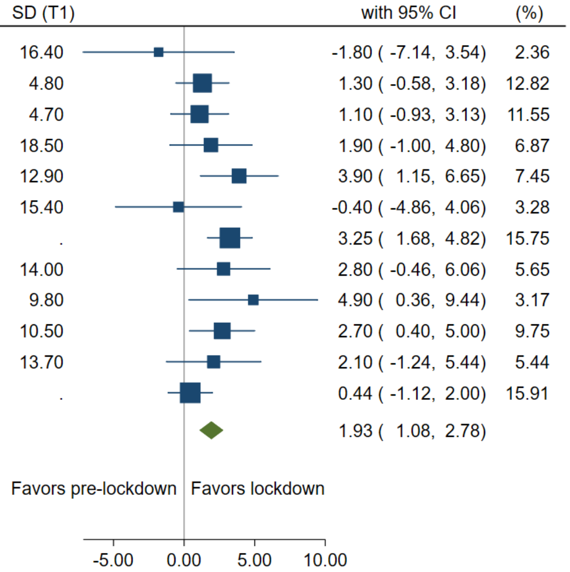

Random-effects REML model

(F) TIR (post-lockdown vs. pre-lockdown)

Study	Total	Mean (T0)	SD (T0)	Mean (T2)	SD (T2)	Effect size with 95% CI	Weight (%)
Di Riso et al. (2021)	71	54.30	13.50	57.00	15.20	2.70 (0.89, 4.51)	24.83
Garza et al. (2022)	143	55.50	16.70	61.30	16.50	5.80 (3.08, 8.52)	19.44
Lombardo et al. (2021)	85	62.70	13.00	66.40	11.70	3.70 (1.06, 6.34)	19.92
Marigliano et al.(2021)	233	52.60	15.20	58.00	15.10	5.40 (3.45, 7.35)	24.01
Wu et al.(2021)	43	52.60	14.40	51.20	15.30	-1.40 (-5.84, 3.04)	11.79
**Overall**						3.67 (1.74, 5.59)	

Heterogeneity: $\tau^2 = 3.02$, $I^2 = 66.10\%$, $H^2 = 2.95$

Test of $\theta_i = \theta_j$: Q(4) = 11.33, p = 0.02

Test of $\theta = 0$: z = 3.74, p = 0.00

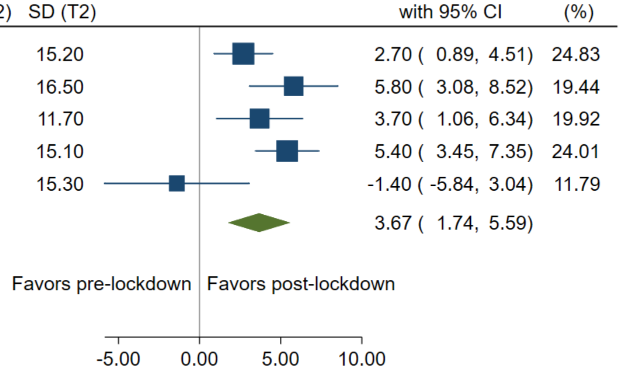

Random-effects REML model

(G) TAR (lockdown vs. pre-lockdown)

Study	Total	Mean (T0)	SD (T0)	Mean (T1)	SD (T1)	Effect size with 95% CI	Weight (%)
Christoforidis et al. (2020)	34	44.90	16.40	47.10	17.30	2.20 (-3.47, 7.87)	4.65
Garza et al. (2022)	143	38.60	16.70	37.60	18.60	-1.00 (-3.91, 1.91)	11.20
Lombardo et al. (2021)	85	33.50	13.40	29.60	13.30	-3.90 (-6.74, -1.06)	11.47
Wu et al.(2021)	43	18.70	4.00	15.40	3.90	-3.30 (-4.48, -2.12)	19.27
Tornese et al. (2020)	13	29.80	4.30	31.00	2.30	1.20 (-0.83, 3.23)	15.09
Tinti et al. (2021)	66	37.80	14.00	35.20	15.00	-2.60 (-6.10, 0.90)	9.12
Schiaffini et al. (2020)	22	34.70	12.80	29.80	10.60	-4.90 (-9.85, 0.05)	5.73
Conejero et al (2022)	80	23.50	9.80	22.00	10.00	-1.50 (-3.67, 0.67)	14.40
Predieri et al.(2020)	62	37.80	13.90	35.70	14.40	-2.10 (-5.62, 1.42)	9.06
**Overall**						-1.84 (-3.21, -0.46)	

Heterogeneity: $\tau^2 = 2.19$, $I^2 = 56.70\%$, $H^2 = 2.31$

Test of $\theta_i = \theta_j$: Q(8) = 20.06, p = 0.01

Test of $\theta = 0$: z = -2.62, p = 0.01

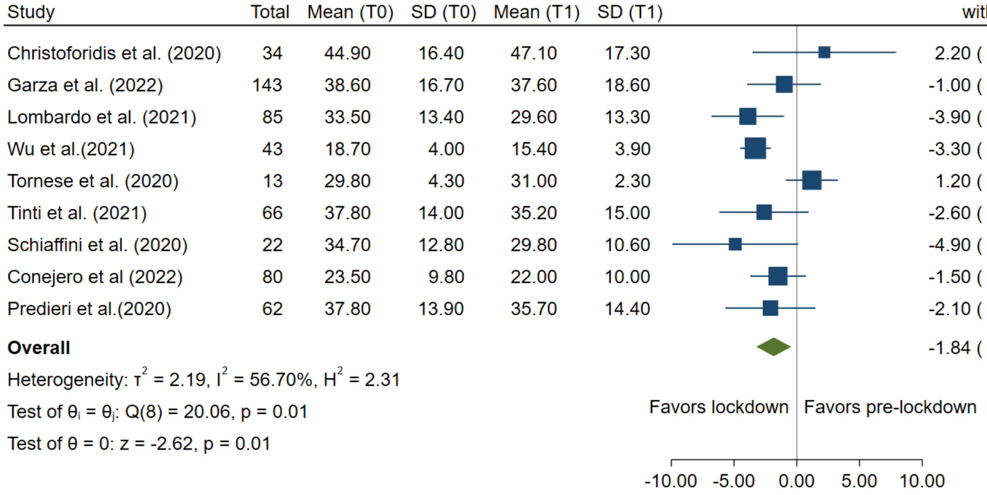

Random-effects REML model

(H) TAR (post-lockdown vs. pre-lockdown)

Study	Total	Mean (T0)	SD (T0)	Mean (T2)	SD (T2)	Effect size with 95% CI	Weight (%)
Di Riso et al. (2021)	71	42.80	14.50	40.20	14.50	-2.60 (-4.64, -0.56)	19.85
Garza et al. (2022)	143	38.60	16.70	33.60	17.10	-5.00 (-7.77, -2.23)	13.09
Lombardo et al. (2021)	85	33.50	13.40	29.40	12.10	-4.10 (-6.82, -1.38)	13.45
Marigliano et al.(2021)	233	43.40	16.20	38.00	15.80	-5.40 (-7.45, -3.35)	19.67
Wu et al.(2021)	43	18.70	4.00	15.80	3.70	-2.90 (-4.05, -1.75)	33.95
**Overall**						-3.77 (-4.93, -2.61)	

Heterogeneity: $\tau^2 = 0.69$, $I^2 = 40.28\%$, $H^2 = 1.67$

Test of $\theta_i = \theta_j$: Q(4) = 6.38, p = 0.17

Test of $\theta = 0$: z = -6.35, p = 0.00

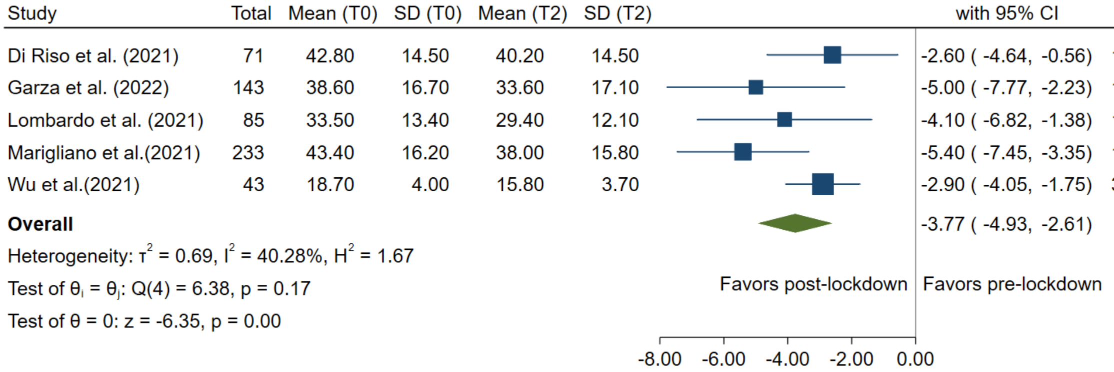

Random-effects REML model

(I) TBR (lockdown vs. pre-lockdown)

Study	Total	Mean (T0)	SD (T0)	Mean (T1)	SD (T1)	Effect size with 95% CI	Weight (%)
Christoforidis et al. (2020)	34	2.60	2.40	2.20	3.30	-0.40 (-1.39, 0.59)	6.47
Garza et al. (2022) MDI	87	7.00	8.80	6.30	9.40	-0.70 (-2.62, 1.22)	2.11
Garza et al. (2022) SCII	63	4.50	3.80	3.40	3.00	-1.10 (-1.96, -0.24)	8.03
Lombardo et al. (2021)	85	3.80	3.50	3.80	3.40	0.00 (-0.73, 0.73)	9.88
Wu et al.(2021)	43	3.70	1.80	2.90	1.10	-0.80 (-1.27, -0.33)	15.77
Tornese et al. (2020)	13	2.00	0.60	1.10	0.80	-0.90 (-1.29, -0.51)	18.04
Tinti et al. (2021)	66	2.50	2.30	2.30	2.50	-0.20 (-0.78, 0.38)	12.94
Schiaffini et al. (2020)	22	4.00	4.40	3.70	3.00	-0.30 (-1.93, 1.33)	2.84
Conejero et al (2022)	80	4.60	3.20	3.20	2.70	-1.40 (-2.05, -0.75)	11.37
Predieri et al.(2020)	62	2.60	2.40	2.10	2.40	-0.50 (-1.10, 0.10)	12.56
**Overall**						-0.67 (-0.96, -0.38)	

Heterogeneity: $\tau^2 = 0.08$, $I^2 = 41.21\%$, $H^2 = 1.70$

Test of $\theta_i = \theta_j$: Q(9) = 13.78, p = 0.13

Test of $\theta = 0$: z = -4.56, p = 0.00

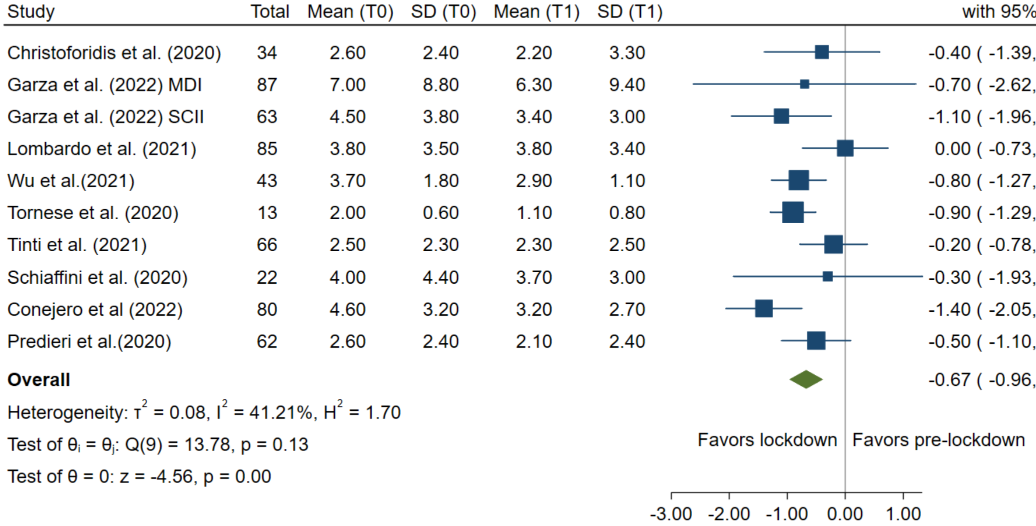

Random-effects REML model

(J) TBR (post-lockdown vs. pre-lockdown)

Study	Total	Mean (T0)	SD (T0)	Mean (T2)	SD (T2)	Effect size with 95% CI	Weight (%)
Di Riso et al. (2021)	71	3.30	2.50	3.10	3.00	-0.20 (-1.18, 0.78)	15.98
Garza et al. (2022) MDI	87	7.00	8.80	7.50	15.40	0.50 (-2.31, 3.31)	4.66
Garza et al. (2022) CSII	63	4.50	3.80	3.60	3.20	-0.90 (-1.77, -0.03)	17.17
Lombardo et al. (2021)	85	3.80	3.50	4.30	3.60	0.50 (-0.25, 1.25)	18.51
Marigliano et al.(2021)	233	2.87	2.49	2.92	2.57	0.05 (-0.27, 0.37)	22.80
Wu et al.(2021)	43	3.70	1.80	5.00	1.80	1.30 (0.76, 1.84)	20.88
**Overall**						0.21 (-0.46, 0.89)	

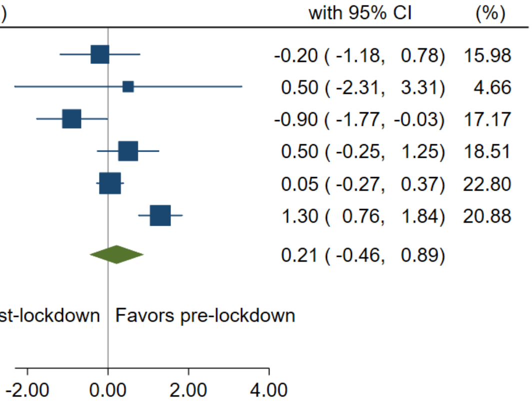

Heterogeneity: $\tau^2 = 0.49$, $I^2 = 80.54\%$, $H^2 = 5.14$

Test of $\theta_i = \theta_j$: Q(5) = 23.99, p = 0.00

Test of $\theta = 0$: z = 0.62, p = 0.54

Random-effects REML model

(K) CV (lockdown vs. pre-lockdown)

Study	Total	Mean (T0)	SD (T0)	Mean (T1)	SD (T1)	Effect size with 95% CI	Weight (%)
Christoforidis et al. (2020)	34	39.50	5.70	37.40	5.90	-2.10 (-4.05, -0.15)	4.49
Dalmazi et al. (2020)- <=12 yrs	30	37.20	1.80	36.60	1.60	-0.60 (-1.21, 0.01)	24.74
Dalmazi et al. (2020)- 13-17 yrs	24	36.70	2.30	35.50	1.80	-1.20 (-2.04, -0.36)	17.28
Lombardo et al. (2021)	85	36.90	6.20	36.00	5.80	-0.90 (-2.18, 0.38)	9.33
Wu et al. (2021)	43	35.50	7.20	34.10	6.50	-1.40 (-3.46, 0.66)	4.08
Tornese et al. (2020)	13	34.60	1.30	34.00	2.30	-0.60 (-1.69, 0.49)	12.03
Tinti et al. (2021)	66	36.00	5.00	35.00	5.00	-1.00 (-2.21, 0.21)	10.22
Conejero et al (2022)	80	35.80	6.30	33.10	6.10	-2.70 (-4.06, -1.34)	8.43
Predieri et al.(2020)	62	36.30	5.30	34.90	4.90	-1.40 (-2.67, -0.13)	9.39
**Overall**						-1.12 (-1.56, -0.69)	

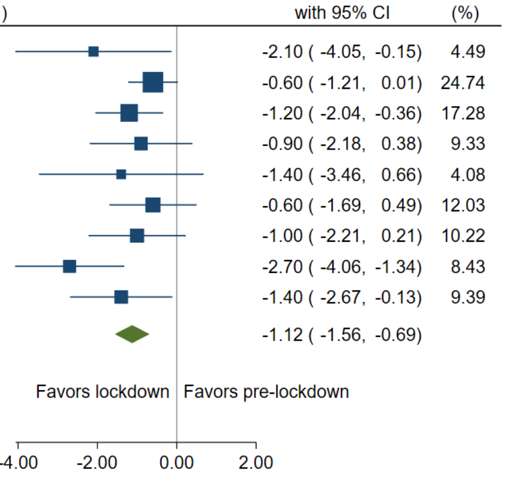

Heterogeneity: $\tau^2 = 0.10$, $I^2 = 23.79\%$, $H^2 = 1.31$

Test of $\theta_i = \theta_j$: Q(8) = 10.10, p = 0.26

Test of $\theta = 0$: z = -5.08, p = 0.00

Random-effects REML model

(L) CV (post-lockdown vs. pre-lockdown)

Study	Total	Mean (T0)	SD (T0)	Mean (T2)	SD (T2)	Effect size with 95% CI	Weight (%)
Di Riso et al. (2021)	71	37.50	4.50	36.70	4.60	-0.80 (-1.86, 0.26)	24.00
Garza et al. (2022)	114	39.60	8.70	37.30	7.40	-2.30 (-3.79, -0.81)	15.30
Lombardo et al. (2021)	85	36.90	6.20	37.10	6.20	0.20 (-1.12, 1.52)	18.22
Marigliano et al.(2021)	233	38.00	5.80	37.50	6.10	-0.50 (-1.26, 0.26)	33.13
Wu et al. (2021)	43	35.50	7.20	35.20	6.40	-0.30 (-2.34, 1.74)	9.34
**Overall**						-0.70 (-1.38, -0.02)	

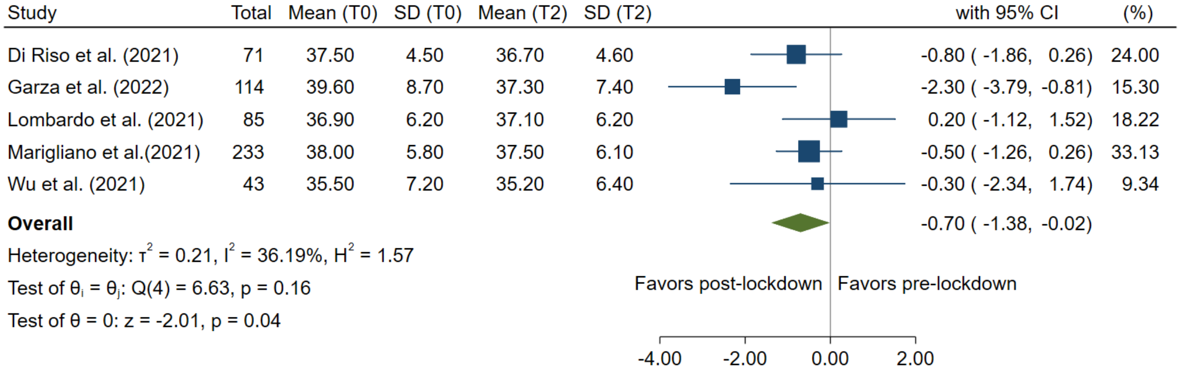

Heterogeneity: $\tau^2 = 0.21$, $I^2 = 36.19\%$, $H^2 = 1.57$

Test of $\theta_i = \theta_j$: Q(4) = 6.63, p = 0.16

Test of $\theta = 0$: z = -2.01, p = 0.04

Random-effects REML model

Supplementary Fig 3. Funnel plots of outcomes which were reported by 10 studies or more.

(A) glucose (lockdown vs. pre-lockdown)

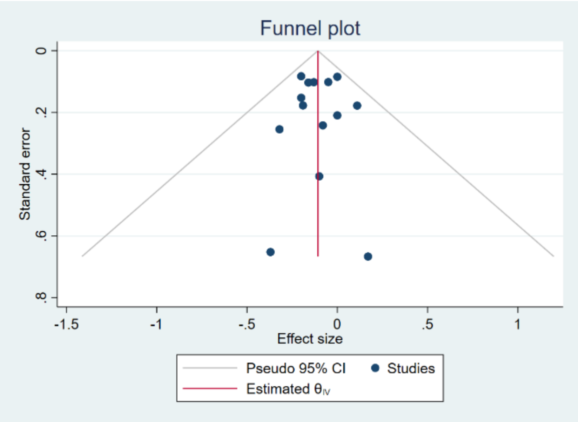

(B) TIR (lockdown vs. pre-lockdown)

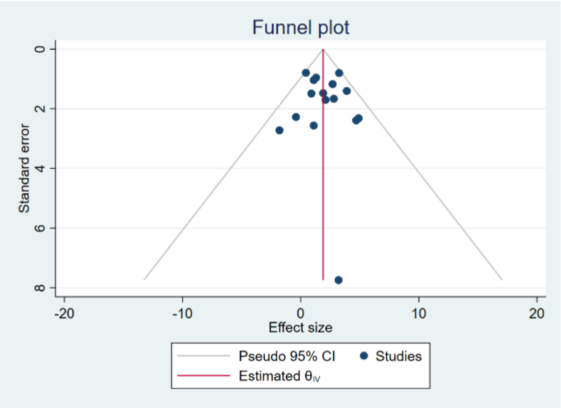

(C) TAR (lockdown vs. pre-lockdown)

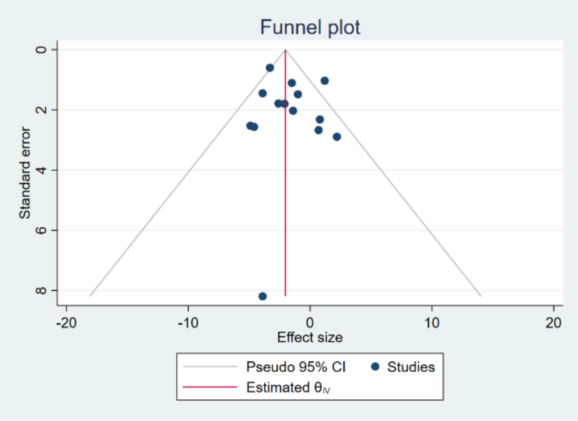

(D) TBR (lockdown vs. pre-lockdown)

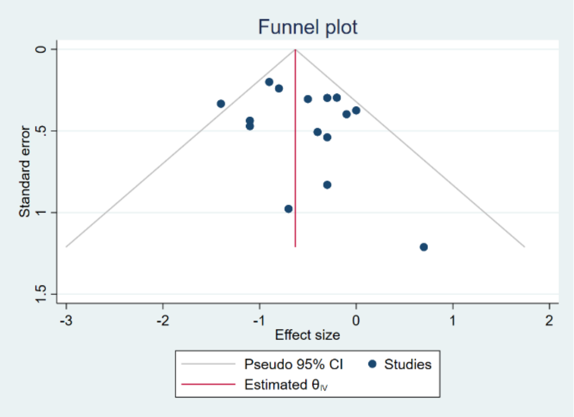

(E) %CV (lockdown vs. pre-lockdown)

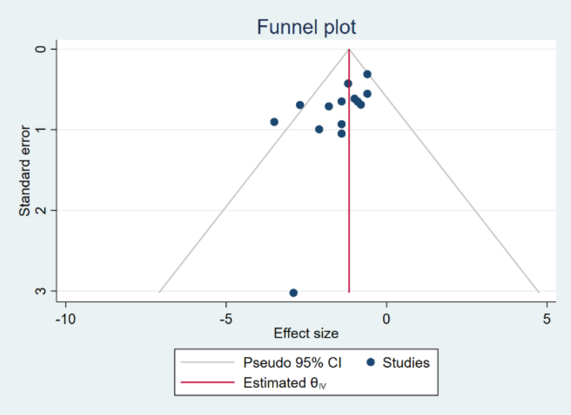

Supplementary Fig 4. Sensitivity leave-one-out analyses.

(A) HbA1c (lockdown vs. pre-lockdown)

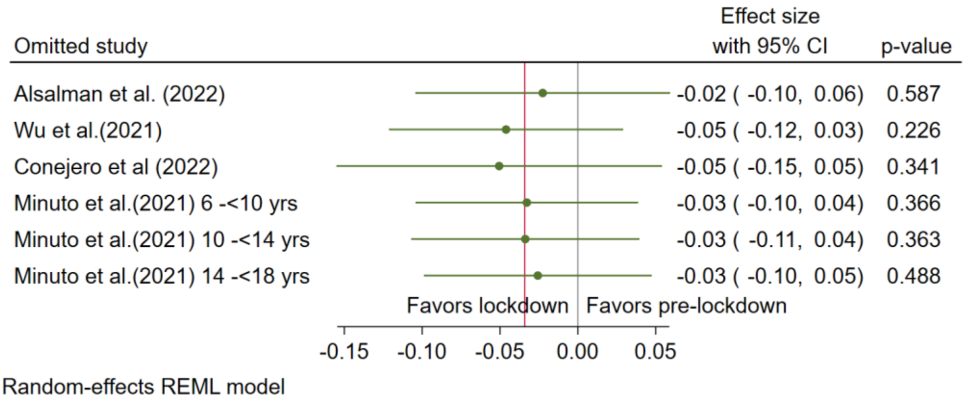

(B) HbA1c (post-lockdown vs. pre-lockdown)

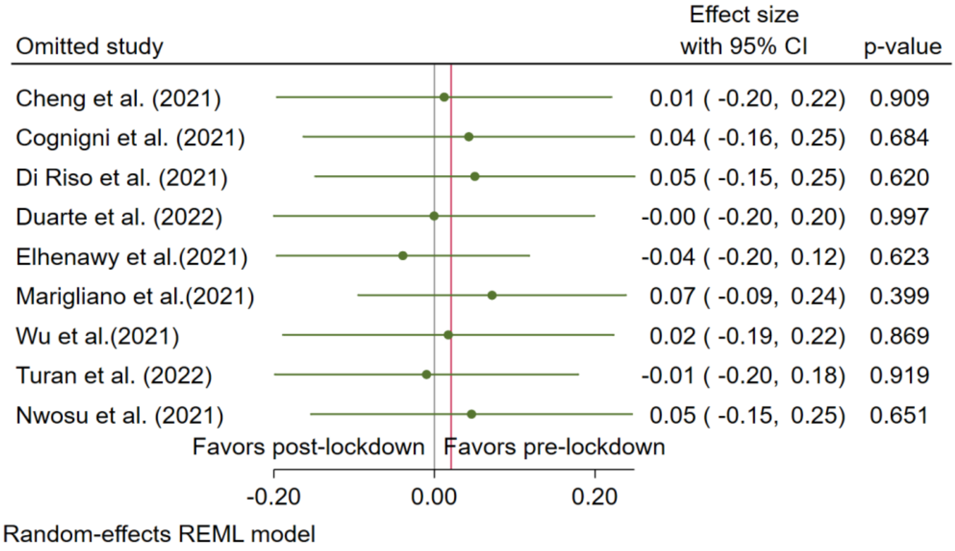

(C) glucose (lockdown vs. pre-lockdown)

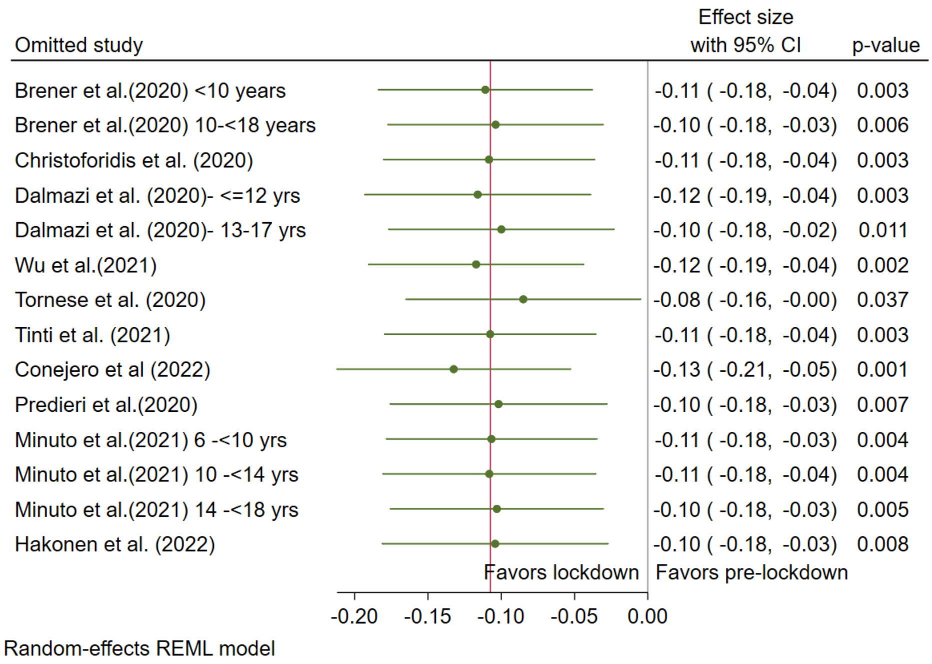

(D) glucose (post-lockdown vs. pre-lockdown)

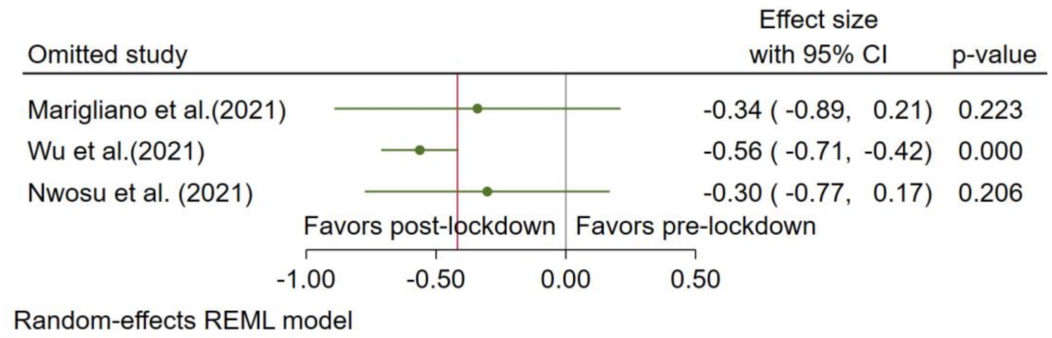

(E) TIR (lockdown vs. pre-lockdown)

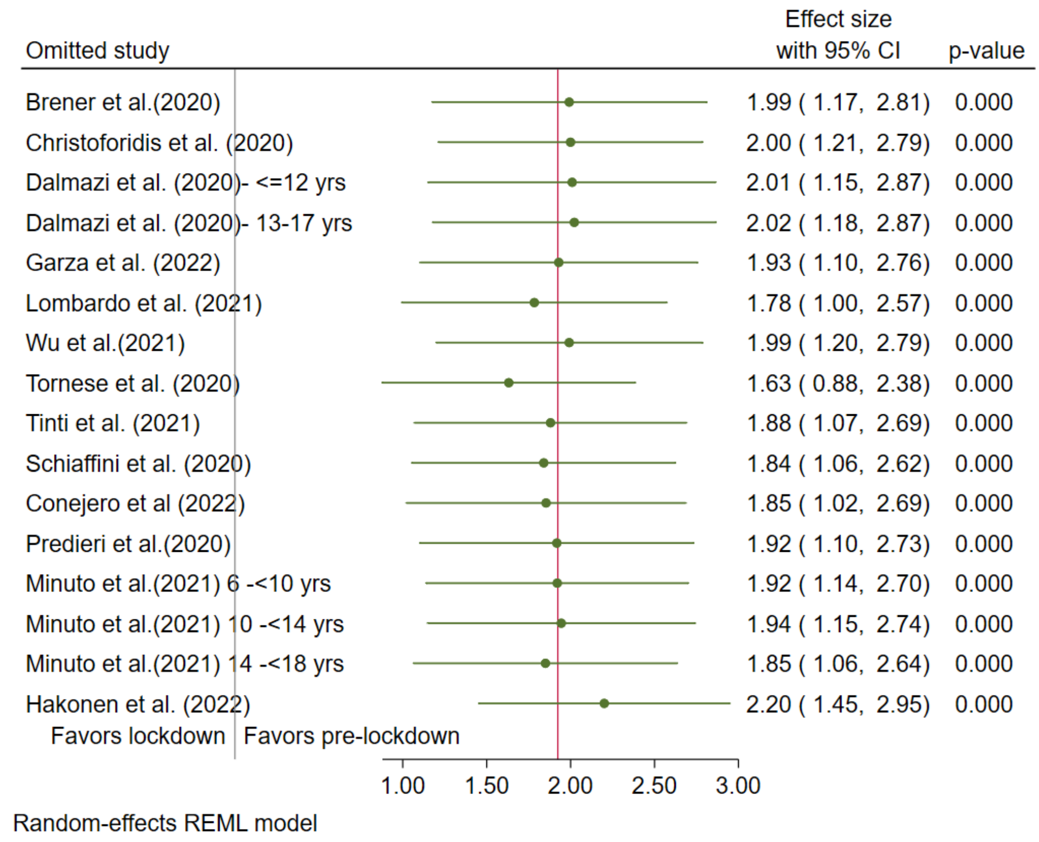

(F) TIR (post-lockdown vs. pre-lockdown)

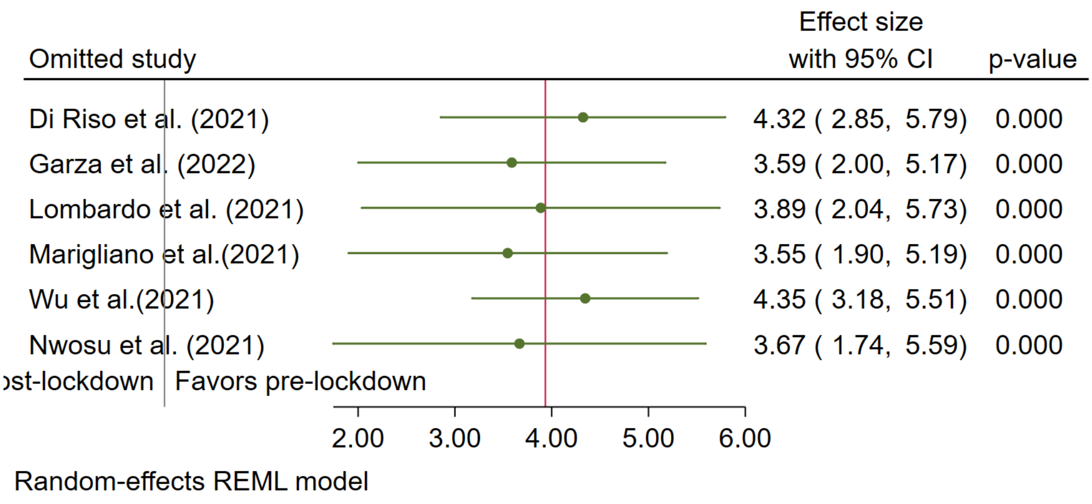

(G) TAR (lockdown vs. pre-lockdown)

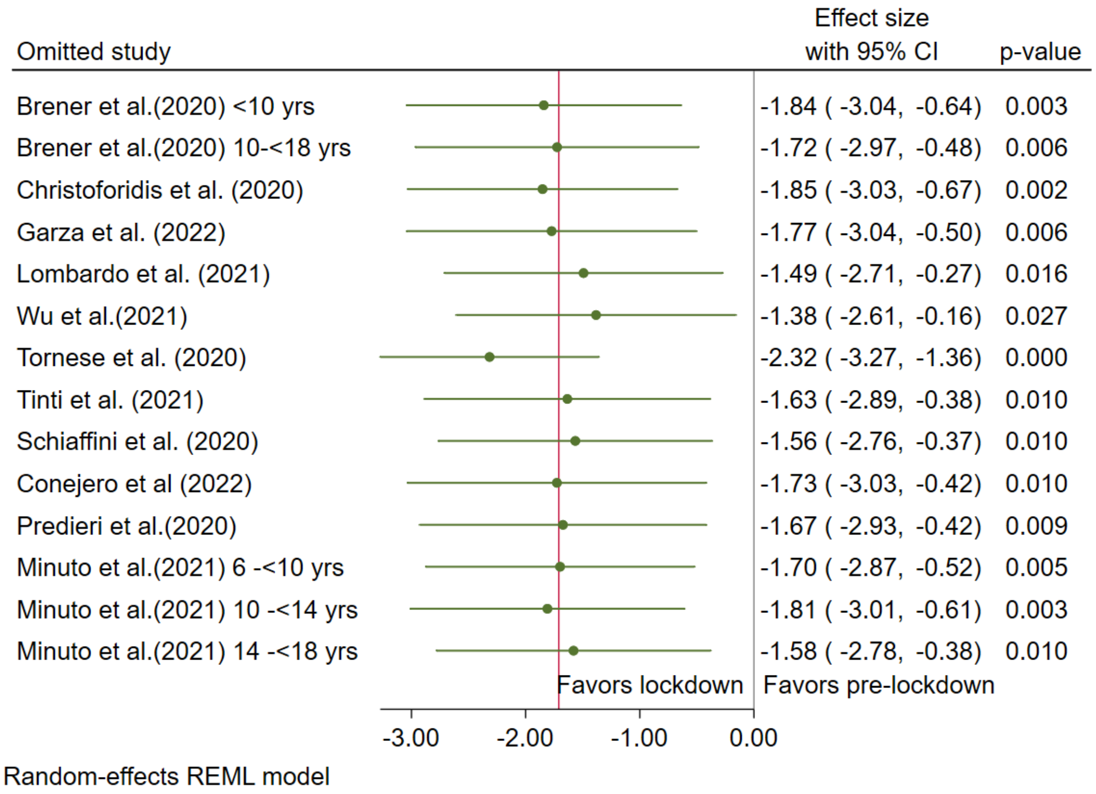

(H) TAR (post-lockdown vs. pre-lockdown)

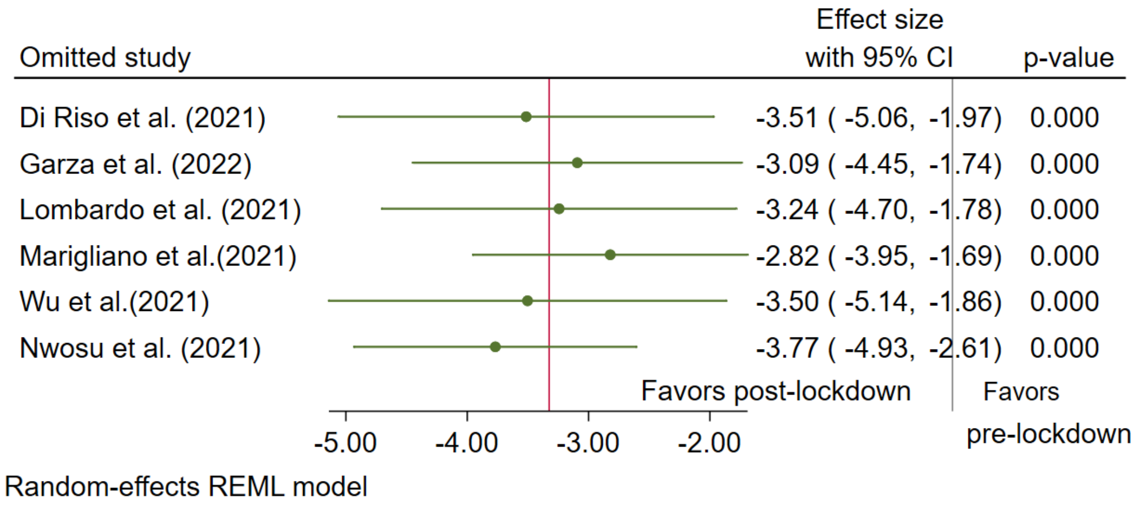

(I) TBR (lockdown vs. pre-lockdown)

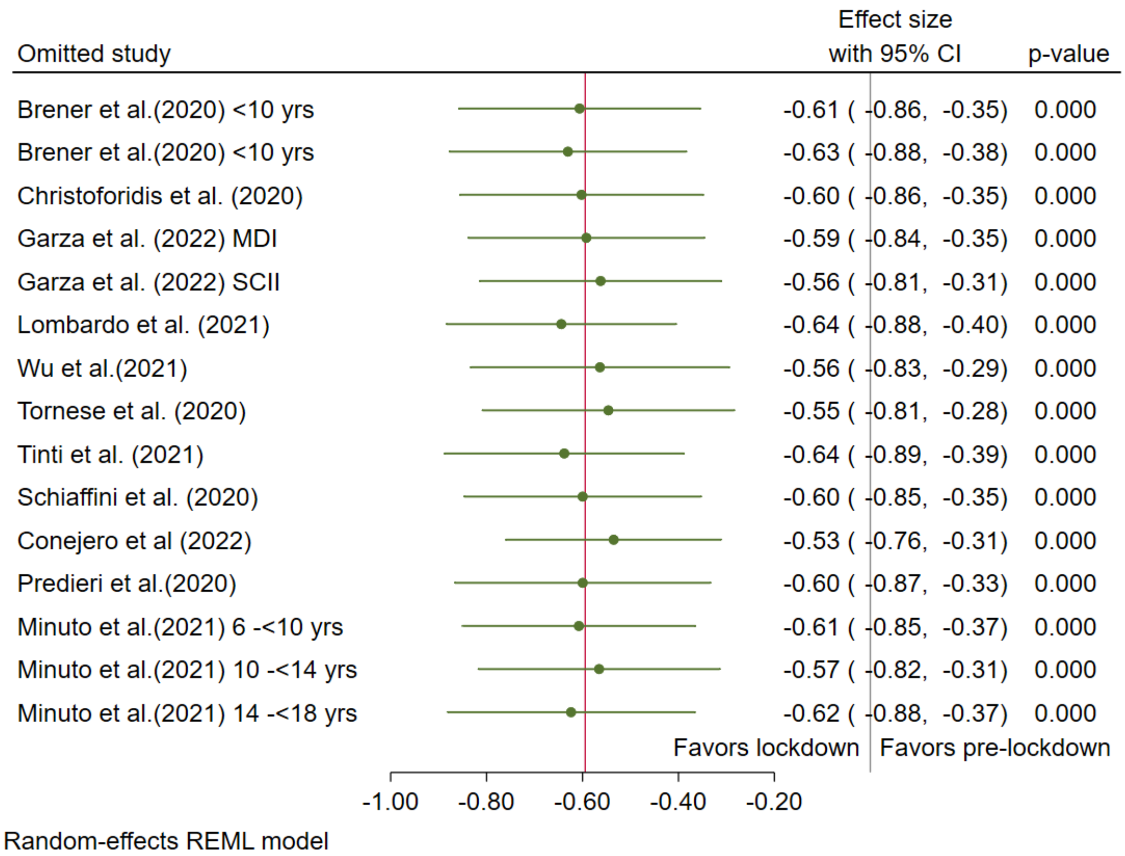

(J) TBR (post-lockdown vs. pre-lockdown)

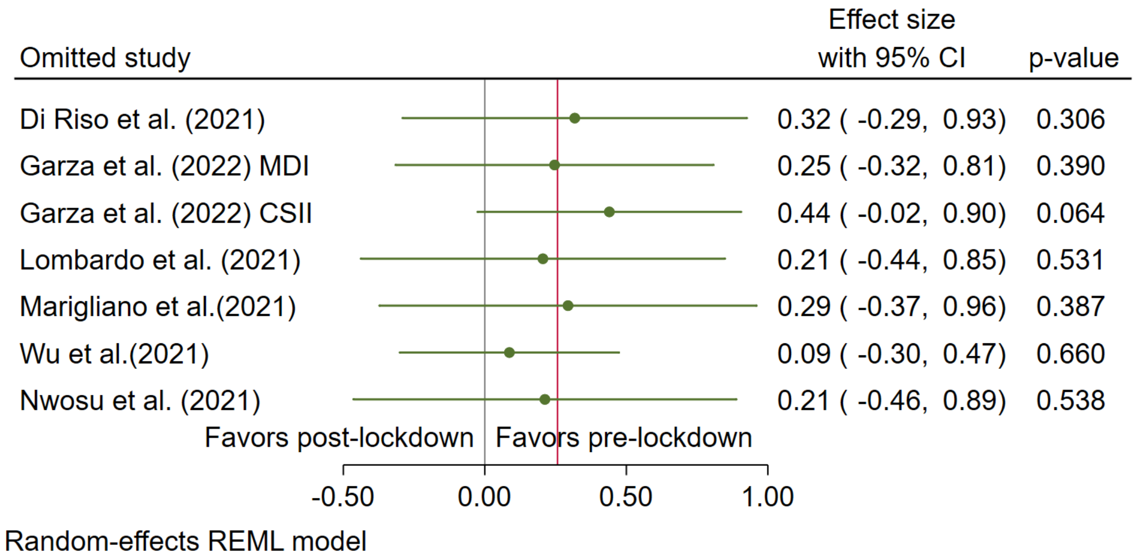

(K) CV (lockdown vs. pre-lockdown)

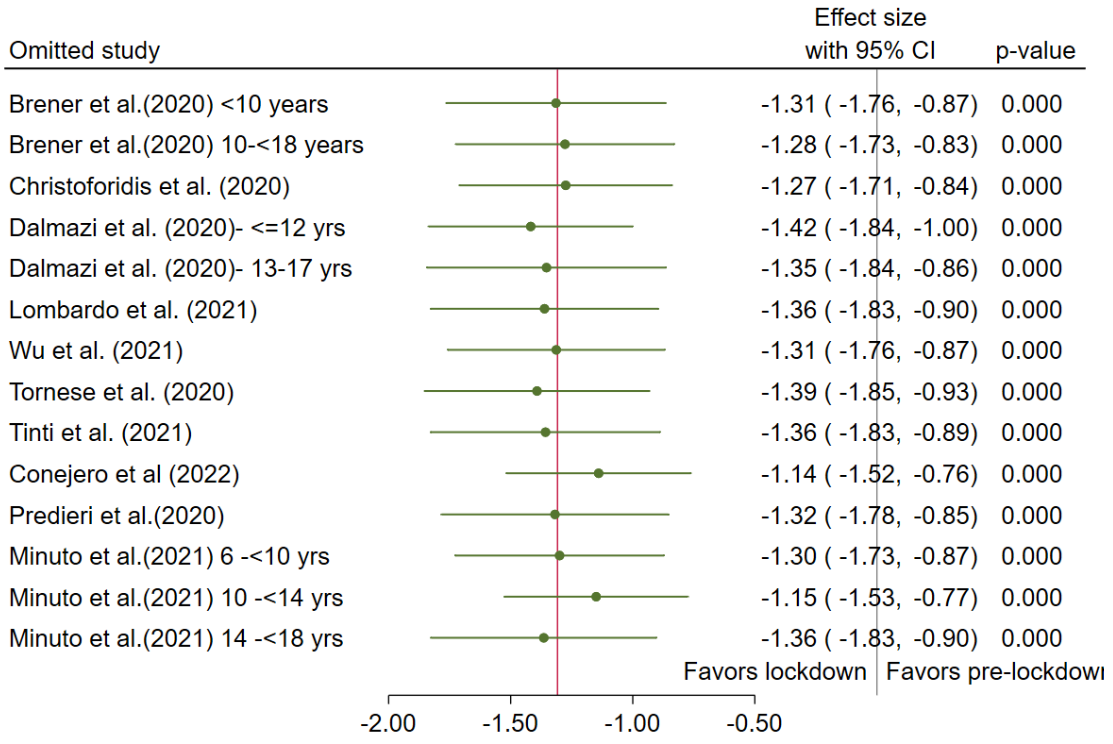

Random-effects REML model

(L) CV (post-lockdown vs. pre-lockdown)

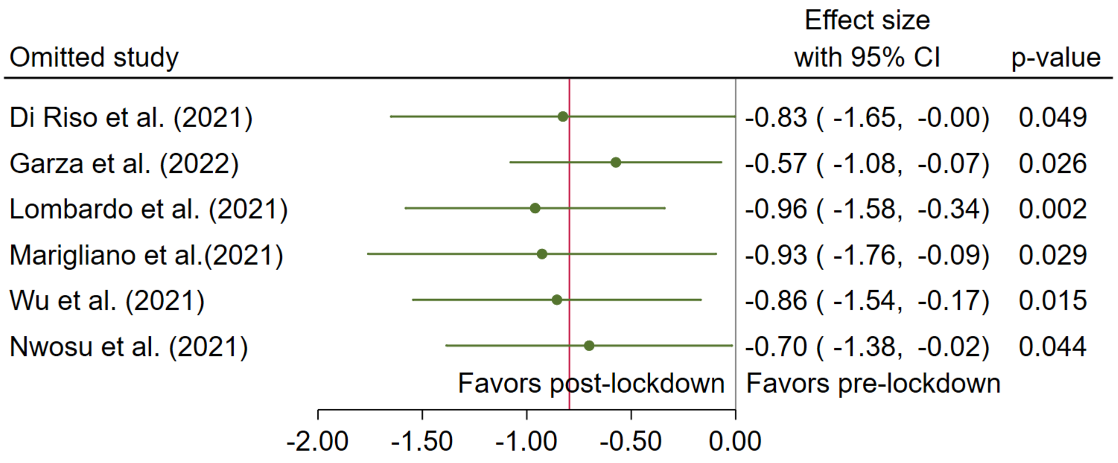

Random-effects REML model
